# Deep Analysis of Mitochondria and Cell Health Using Machine Learning

**DOI:** 10.1038/s41598-018-34455-y

**Published:** 2018-11-05

**Authors:** Atena Zahedi, Vincent On, Rattapol Phandthong, Angela Chaili, Guadalupe Remark, Bir Bhanu, Prue Talbot

**Affiliations:** 1 Graduate Program in Bioengineering, University of California, Riverside, CA. USA; 2 Department of Electrical & Computer Engineering, University of California, Riverside, CA. USA; 3 Department of Molecular, Cell and Systems Biology, University of California, Riverside, CA. USA; 4 Department of Computer Science, University of California, Riverside, CA. USA

## Abstract

There is a critical need for better analytical methods to study mitochondria in normal and diseased states. Mitochondrial image analysis is typically done on still images using slow manual methods or automated methods of limited types of features. MitoMo integrated software overcomes these bottlenecks by automating rapid unbiased quantitative analysis of mitochondrial morphology, texture, motion, and morphogenesis and advances machine-learning classification to predict cell health by combining features. Our pixel-based approach for motion analysis evaluates the magnitude and direction of motion of: (1) molecules within mitochondria, (2) individual mitochondria, and (3) distinct morphological classes of mitochondria. MitoMo allows analysis of mitochondrial morphogenesis in time-lapse videos to study early progression of cellular stress. Biological applications are presented including: (1) establishing normal phenotypes of mitochondria in different cell types; (2) quantifying stress-induced mitochondrial hyperfusion in cells treated with an environmental toxicant, (3) tracking morphogenesis in mitochondria undergoing swelling, and (4) evaluating early changes in cell health when morphological abnormalities are not apparent. MitoMo unlocks new information on mitochondrial phenotypes and dynamics by enabling deep analysis of mitochondrial features in any cell type and can be applied to a broad spectrum of research problems in cell biology, drug testing, toxicology, and medicine.

## Introduction

Mitochondria are dynamic organelles capable of regulating cell fate, homeostasis, survival, and disease in eukaryotic cells^1–3^. Mitochondrial phenotypes (morphology, dynamics, and organizational patterns) vary significantly in different cell types. During fusion and fission^4^, mitochondria transition between morphological classes that include small puncta, tubes, networks, and “donuts” or rings^5,6^. These morphologies are related to the metabolic state and bioenergetics of the cell and vary during processes such as cell division and differentiation^3,7^. Mitochondria have an intrinsic ability to sense their state of health, and when stressed, induce compensatory quality-control mechanisms, such as stress-induced mitochondrial hyperfusion (SIMH) or fission and degradation of damaged mitochondria (mitophagy)^6,8–10^, making them excellent organelles for evaluating cell health. Moreover, mitochondrial morphology and dynamics are altered in common neurodegenerative diseases, such as Alzheimer’s disease (AD), Parkinson’s disease (PD), amyotrophic lateral sclerosis (ALS), and Huntington’s disease (HD)^11^ and may vary within subclasses of diseases such as cancer, diabetes, myopathies and metabolic diseases^7,11–14^. For example, changes in mitochondrial morphology, mainly fragmentation, and abnormal dynamics in axonal transport in neurons have been reported in HD patients^11^. In diseases such as cancer, mitochondria phenotypes have been shown to vary between tumors, and used to classify types of cancer^15,16^.

Because of their importance in homeostasis, stress, and human disease, there is need for technologies to analyze and quantify changes in mitochondrial morphology and dynamic behavior. Time-consuming manual protocols^17^ are being replaced by software that provides automated analysis of mitochondrial features, making rapid high content analysis feasible. While mitochondrial analysis software is continually evolving, some existing programs have limitations with respect to accessibility. Some require that users know programming languages and have access to commercial image processing software not routinely available in all labs^18,19^. In this paper, we introduce MitoMo, which is open-source, provides a user-friendly graphical user interface (GUI) that does not require programming knowledge, can easily be adapted to any laboratory, and is flexible in allowing users to import pre-segmented images from any image processing software.

Because of limitations in existing software, there is an unmet need for software that can perform an integrated multi-feature analysis of morphology, motion, texture, and morphogenesis. While most software provide segmentation, feature extraction, and classification modules, they are limited in their image processing^15,20^ and types of feature analysis^15,16,18–23^. Our software provides users with additional pre-processing (histogram matching, tophat) and post-segmentation (declumping, morphological operations) steps, which significantly improve the accuracy of segmentation. Most software use one type of classification algorithm (typically a decision tree type)^15,18,23^ and are capable of only mitochondrial morphology analysis or cell classification. MitoMo provides users with multiple classification algorithms and performs both morphological and cell health classification. MitoMo can perform on multiple scales, enabling the study of individual mitochondria, patches of mitochondria, or mitochondrial populations in entire cells. It also divides feature data across the morphological classes of mitochondria to investigate the contribution of each class to an experimental stimulus or disease.

Mitochondrial morphology and dynamics are both coupled to mitochondrial function^12,24^, stress^8,9,25^, and disease^1,11,13,14^. Previous software have studied motion of individual mitochondria, such as their movement toward regions of energy demand^26^. Our novel intensity flow method^27^ can study sub-organelle motion, which relates to the flow of molecules within the mitochondria, a type of motion has rarely been studied. Motion analysis was further expanded in MitoMo to include directionality with respect to any cellular structure. This reveals organizational changes of mitochondria inside the cell, which correlate to changes in energy demand or association with other cellular structures (e.g., endoplasmic reticulum, autphagosomes, etc). Lastly unlike other mitochondrial based software, MitoMo can be used to analyze video data, thereby providing morphological information on mitochondrial morphogenesis during cell differentiation, toxicant treatment, or disease progression.

Our purpose is to introduce MitoMo an open-source, user-friendly software that integrates multiple feature types and goes beyond existing software to enhance mitochondrial analysis and cell health classification. Several applications of the MitoMo are presented to demonstrate its broad potential in cell and medical research, drug development, and toxicology.

## Results

### MitoMo integrated pipeline

**Figure 1:**
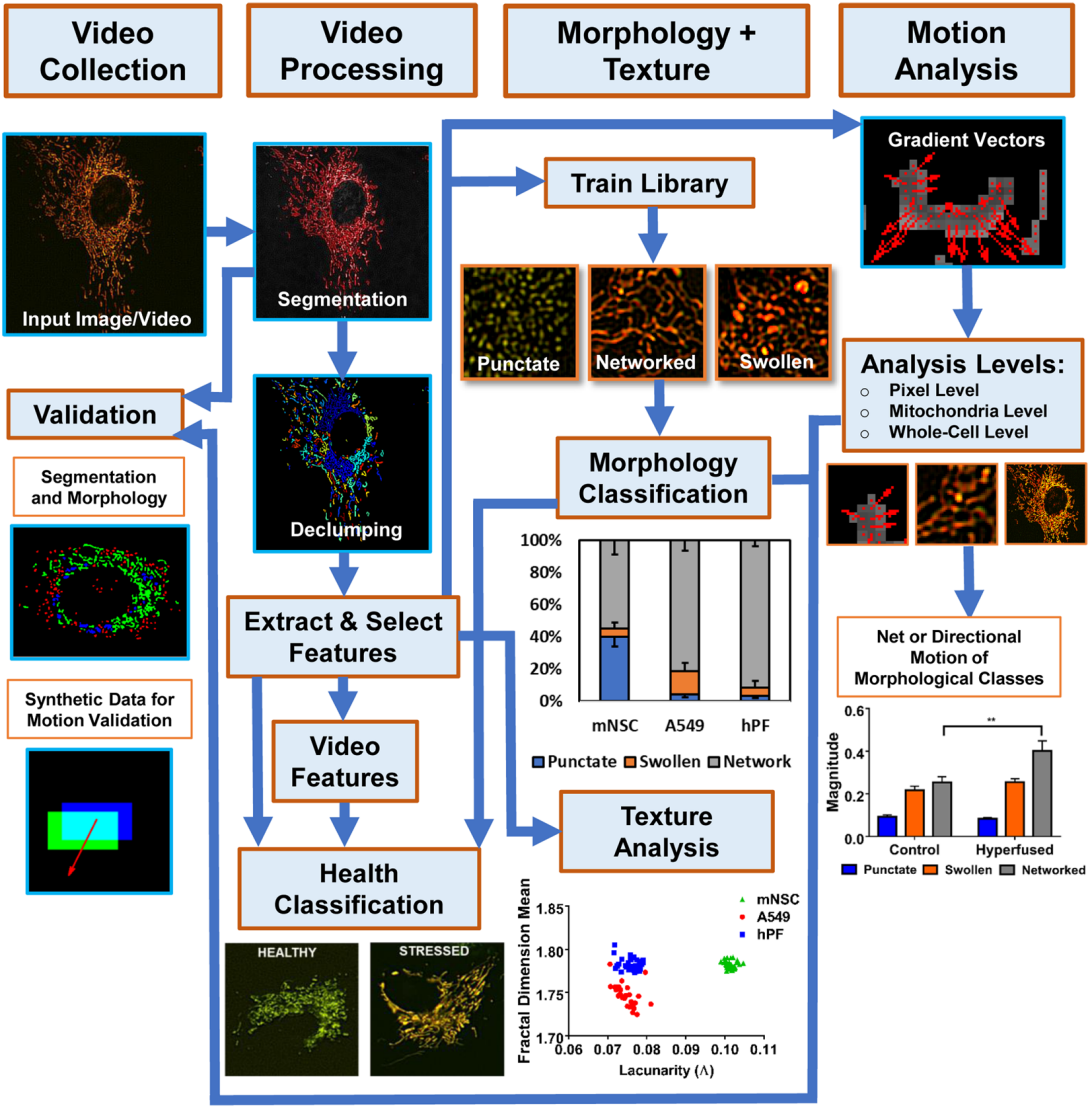
Overview of MitoMo software pipeline. Video frames of labeled mitochondria were captured and loaded into the MitoMo software. The videos were segmented and declumped, and 21 morphological, intensity, texture, and motion features were extracted for each frame. The selected features were then fed into a previously trained library of the three mitochondrial morphologies (punctate, networked, and swollen). The segmented mitochondria from the test videos (frames) were automatically morphologically classified using K-nearest neighbor (KNN) and Naïve Bayes. The resulting data were plotted into graphs, depicting the percentage of punctate, networked, and swollen mitochondria. The software can also perform motion analysis by computing the magnitude and orientation of the gradient vectors, and the net or directional motion can be plotted for the population of mitochondria in each cell. Motion analysis can be performed for the entire mitochondrial population within a cell, individual mitochondria, and/or morphological classes of mitochondria. Texture features, which are indicative of mitochondrial complexity and organization, can be extracted, Validation was performed to ensure accuracy of the segmentation, morphological classification, and motion analysis. Extracted features can be combined to perform health classification during low-level stress that is otherwise not detectable.

MitoMo is an automated image/video processing and machine-learning software that is designed to work with fluorescent images. In the MitoMo pipeline (Fig. 1), still or time-lapse live-cell videos were collected from three different cell types that were transiently or stably transfected with MitoTimer, a mitochondria-targeted fluorescent reporter that enables quantification of mitochondrial protein oxidation. Any mitochondrial-targeted dye or reporter could be used with MitoMo to quantify functional readouts. Based on user preference, mitochondria can be segmented directly using global or adaptive thresholding and de-clumping procedures in MitoMo, or alternatively, images previously segmented with other software, such as CellProfiler^28^, can be imported into the MitoMo graphical user interface (GUI). To train MitoMo for classification of mitochondrial morphology, features (listed in Methods) were extracted from individual punctate, networked, or swollen mitochondria. Segmented mitochondria were then automatically classified using machine-learning K-nearest neighbor (KNN)^29^ and Naïve Bayes algorithms^30^. In the computational analysis of motion, the density of fluorescently-tagged mitochondrial proteins was assumed to be proportional to the pixel intensity. By computing the change in intensity between adjacent frames, the flow of fluorescently-tagged mitochondrial proteins can be estimated and summed up at the individual pixel level, mitochondrial level, and whole-cell level. The extracted features are used to generate the motion gradient vectors at each individual pixel, and the net sum of the gradient vectors (magnitude) and their direction (orientation) can be plotted as outputs for the motion analysis. Texture features can also be used to investigate the organizational complexity (dense versus porous phenotype) and compactness of the mitochondria within cells. MitoMo multiplexes extracted features and machine learning methods to allow for high-throughput, unbiased, and time/resource-saving characterization and classification of mitochondrial health in any cell type.

### Validation of MitoMo

**Figure 2:**
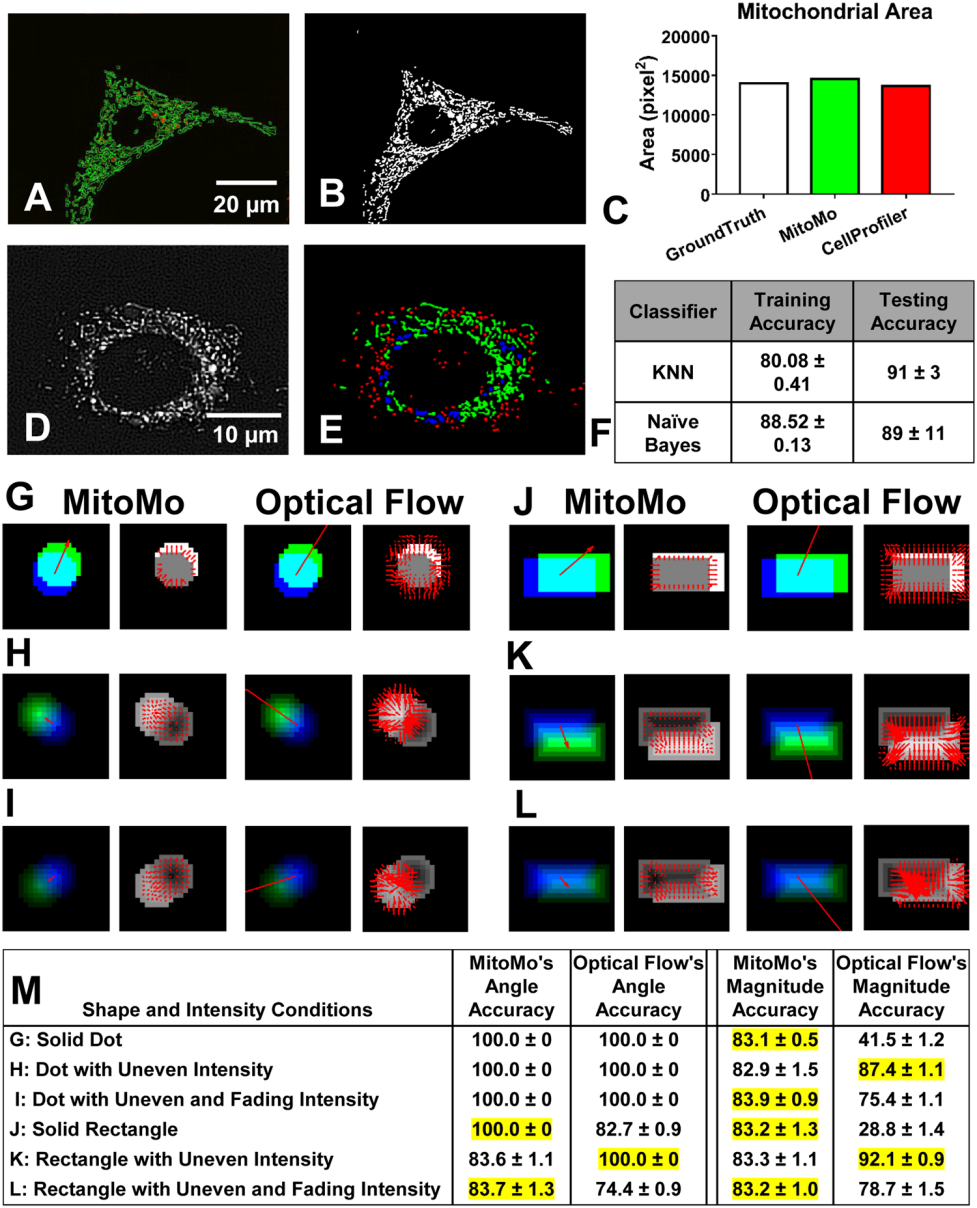
MitoMo validation and comparison of our motion analysis to optical flow. Segmentation validation: (**A**) Manually drawn segmentation (green) using ImageJ was compared to (**B**) segmentation performed by MitoMo. (**C**) The segmentation accuracy (area of overlaps) of the manually labeled versus automatically segmented mitochondria (MitoMo and CellProfiler) were not significantly different using one-way ANOVA with Dunnett’s post hoc test. (**D**–**E**) The morphological classification was validated by comparing manually labeled mitochondria (**D**) against automatic classification (**E**). (**F**) There was up to 88% accuracy with Naïve Bayes and 80% accuracy with KNN in the automatic classification of mitochondrial morphology in 1761 trials using the training data. There was up to 89% accuracy with Naïve Bayes and 91% with KNN in the automatic classification of the test set. (**G**–**L**) Images showing motion analysis validation for both magnitude (blue, green cyan images) and direction (images with red arrows). Each magnitude image is a synthetic image of unique shape and change in intensity overlaid with a motion vector. Blue is object from frame 1, green is object from frame 2, and cyan is overlap of the two frames. Column 1 in (**G**–**L**) shows summed MitoMo vector (indicated by arrow) overlaid onto color coded image. Column 2 is the normalized difference image with MitoMo motion vectors. Column 3 shows the summed Lucas-Kanade vector (indicated by arrow) overlaid onto the color-coded images. Column 4 is the normalized difference image with Lucas-Kanade motion vectors. (**G**) Solid dot with no change in intensity. (**H**) Dot with uneven intensity. (**I**) Disc with uneven intensity that fades to half the brightness by the second frame. (**J**) Solid rectangle with no change in intensity. (**K**) Rectangle with uneven intensity. (**L**) Rectangle with uneven intensity that fades to half the brightness by the second frame. (**M**) Table summarizes the accuracy of angle and magnitude for MitoMo versus optical flow. The angle and magnitude accuracies were statistically compared using t-test and Chi-squared tests, and the more accurate software was highlighted in yellow.

Segmentation and morphological classification, as performed by MitoMo, were rigorously validated. To assess accuracy, segmentation was manually drawn using ImageJ (Fig. 2A) and compared to segmentation performed by MitoMo (Fig. 2B). The percent overlay of the ground-truth segmentation versus the segmentation derived with MitoMo and/or CellProfiler was not statistically different (Fig. 2C). Morphological classification ground-truth was compared to the software’s automatic classification (2D,E). Classification using eight features (Area, Major Axis, Minor Axis, Solidity, Perimeter, Max Radius, Median Radius, Integrated Intensity) resulted in 88% accuracy with Naïve Bayes and 80% accuracy using KNN with five neighbors on the training set. Classification accuracies were 89% with Naïve Bayes and 91% with KNN on the testing set (Fig. 2F).

MitoMo’s motion algorithm was validated and compared against optical flow^31^, which is a representation of the apparent motion of objects, surfaces, edges, and pixels in an image. Optical flow is commonly used for motion estimation to describe the transformation of one frame to another. In the calculation of optical flow, a major assumption is a brightness constraint that requires the appearance or brightness pattern of an object to be constant over a delta change in time^32^. MitoMo does not have this constraint because it estimates the change of intensity between frames and not the motion of specific pixels. To validate our motion analysis, synthetic motion data of various shapes were generated (Fig. 2G–L). For both methods, all generated vectors are summed to produce a single vector estimating the motion of the object. In the table in Fig. 2M, MitoMo had angle accuracies ranging from 83 to 100% and magnitude accuracies ranging from 82 to 83%. MitoMo’s angle and magnitude estimation performed as well or better than the Lucas-Kanade optical flow method in all cases except the uneven intensities, which better satisfy the brightness assumptions of optical flow. Two-tailed t-tests and Chi-squared tests (in cases with no variance) were performed for each comparison, revealing statistically significant improvements in accuracy with MitoMo’s motion analysis in most cases (Fig. 2M). Further details are provided in the Supplementary Data #2.

### Establishing baseline phenotypes of mitochondria in healthy cells

**Figure 3:**
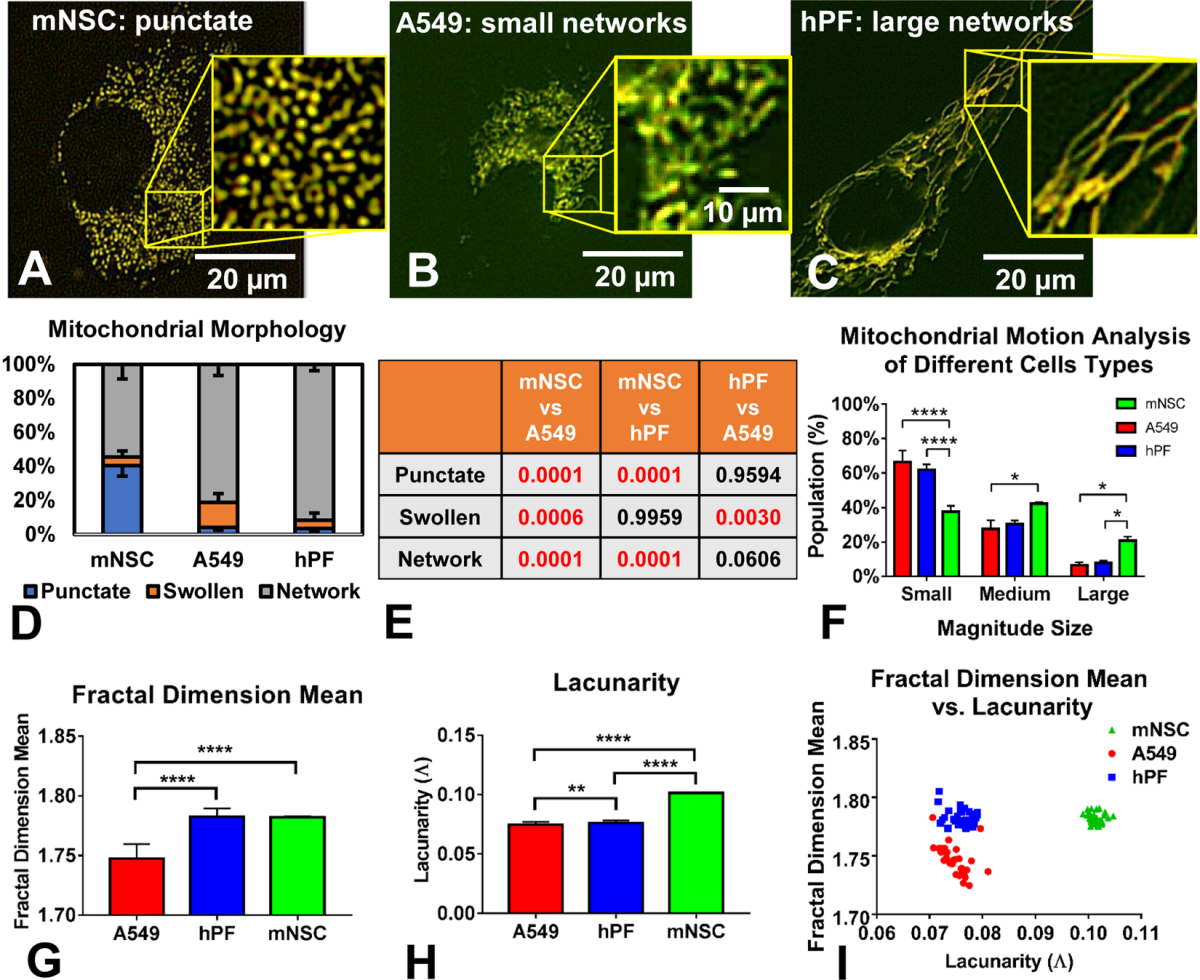
Analysis of mitochondrial phenotype (morphology and texture) in three cell types. (**A**–**C**) Mitochondrial morphology in NSC, human A549 lung epithelial cells, and hPF during normal culture conditions. (**D**) A549 and hPF cells had significantly more networked mitochondria than the NSCs. (**E**) The table summarizes the p-values for each of the morphological groups compared across the different cell types in (**D**) (1-way ANOVA with Tukey’s multiple comparisons test). (**F**) Total motion analysis showed a significant increase in mitochondrial motion in the NSC compared to the other two cell types, which had similar motion profiles (2-way ANOVA with Tukey’s multiple comparisons test). (**G**) Mean fractal dimension analysis showed a lower complexity level in the A549 mitochondria. (**H**) Lacunarity analysis showed a denser mitochondrial organization in the NSCs, which contain a high number of punctate mitochondria. Texture data were analyzed using a 1-way ANOVA with Bonferroni’s multiple comparisons test. (**I**) Fractal dimension mean plotted versus lacunarity for the three cell types showed three distinct clusters.

We define phenotype to incorporate mitochondrial morphology, motion, texture, and morphogenesis. The phenotype and number of mitochondria in a cell depends on the metabolic requirements of the cell, and number may vary from one to thousands across different cell types^7^. MitoMo was first used to establish the mitochondrial phenotypes in three healthy cell types from two species: mouse neural stem cells (NSC), human lung cancer cells (A549), and human primary lung fibroblasts (hPF) (Fig. 3A–C). The cells were transfected with the MitoTimer reporter and imaged live 24 hours later. While all cell types had punctate, networked, and swollen mitochondria, the percentages of each class of mitochondrion were significantly different in each cell type (Fig. 3D,E). Figure 3E summarizes the p-values for each of the morphological groups compared across the different cell types using a 1-way ANOVA followed by Tukey’s post hoc test.

The magnitude of motion of all the mitochondria in the three cell types was calculated (Fig. 3F) and compared statistically across cell types using a 2-way ANOVA with Tukey’s multiple comparisons test. Motion in the A549 cells and hPFs, which had mainly networked mitochondria, was similar, while the punctate mitochondria in the NSCs had significantly elevated motion. The increase in motion in these healthy NSCs is directly correlated with mitochondrial morphology.

To further assess the organization of mitochondria in different cell types, texture features were analyzed. The mean fractal dimension was computed to measure the level of mitochondrial complexity at the whole-cell level (Fig. 3G), using a 1-way ANOVA with Bonferroni’s post hoc test. In NSCs, the small punctate mitochondria are slightly variable in shape and not evenly spaced, which increased complexity and resulted in a higher fractal dimension. Similar to NSCs, the fractal dimension was also high in hPFs, since the highly meshed networks create a higher level of complexity. In contrast, the mesh-work of mitochondria in A549 was more uniformly spaced, resulting in the lowest mean fractal dimension value.

Another texture feature, lacunarity, was compared across the three cell types using a 1-way ANOVA with Bonferroni’s post hoc test (Fig. 3H). Patterns having more or larger gaps or more heterogeneity generally have high lacunarity. This analysis showed significantly higher lacunarity in mNSC than in the other two cell types. Fractal dimension mean versus lacunarity values were plotted for all cell types, revealing distinct, well-separated clusters between the three cell types (Fig. 3I). This shows that texture features are another excellent means of phenotyping mitochondria in different cell types and by extension in different experimental conditions.

### MitoMo detects both directional motion and changes in direction

**Figure 4:**
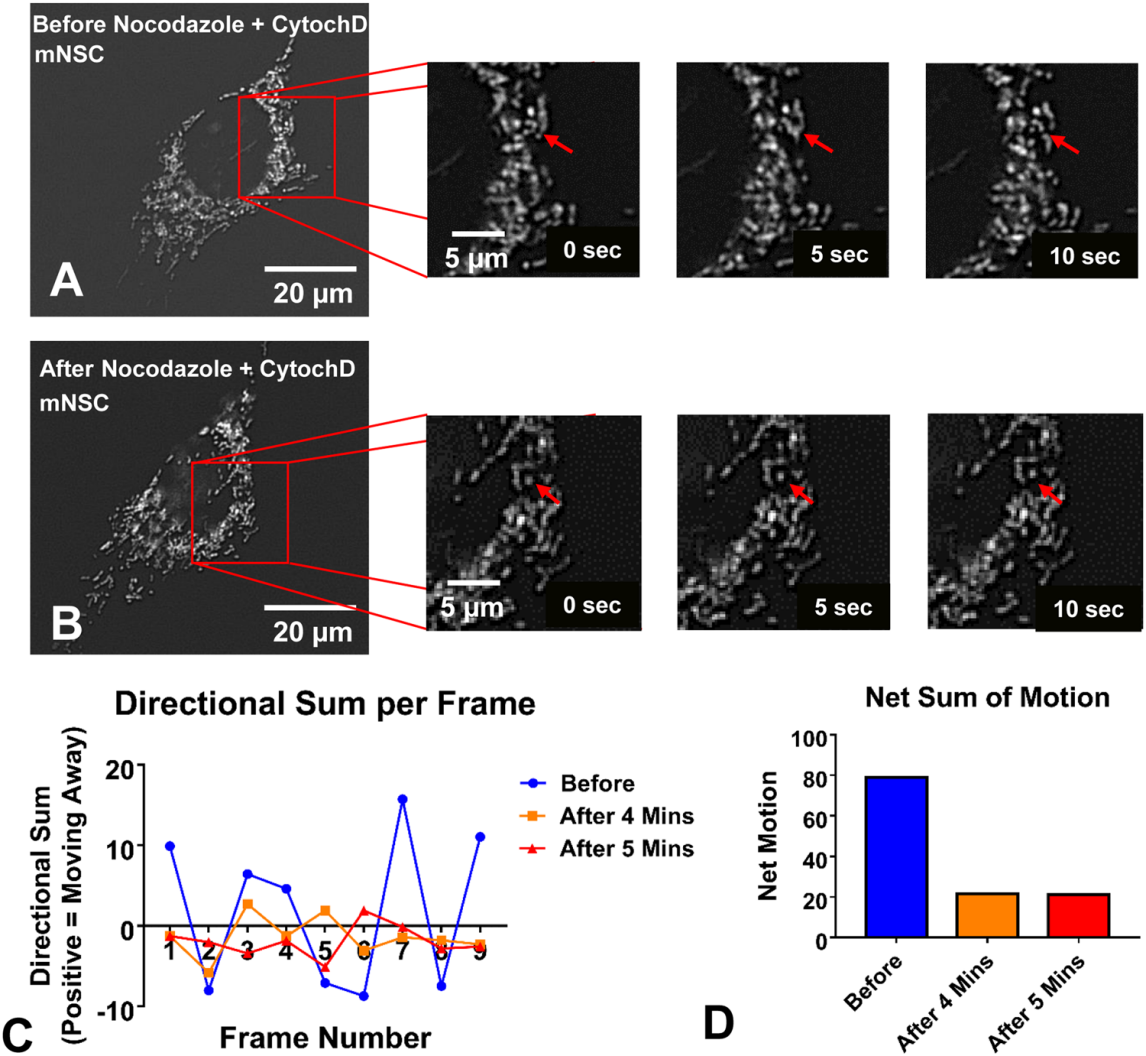
Directional motion analysis in living cells. (**A**,**B**) Images collected before and 4–5 minutes after the addition of nocodazole and cytochalasin D to destabilize the cytoskeleton. (**C**) MitoMo computed random localized motion in the control videos before treatment and showed that movement was arrested after treatment. (**D**) The net sum of motion was quantified over the entire video, showing a decrease in motion after 4 and 5 minutes.

MitoMo has the ability to compute directional motility, in which a reference point, such as the nucleus is chosen, and changes in direction are obtained. To demonstrate the ability of MitoMo to detect motion features, NSC were treated with nocodazole and cytochalasin D to disrupt the microtubules and actin cytoskeleton, respectively, and 10 second live videos were collected (Fig. 4A,B). Motion analysis of the entire mitochondrial population was conducted before treatment and 4 and 5 min after treatment. The directional component of the motion away or towards the nucleus was plotted over time (video frames) revealing fluctuating localized motion in the control videos (before treatment) and arrested movement 4 and 5 min after treatment (Fig. 4C). The net sum of motion over all frames when quantified showed a decrease in motion after 4 and 5 min of treatment (Fig. 4D).

### Morphology and motion analysis following stress-induced mitochondrial hyperfusion (SIMH)

**Figure 5:**
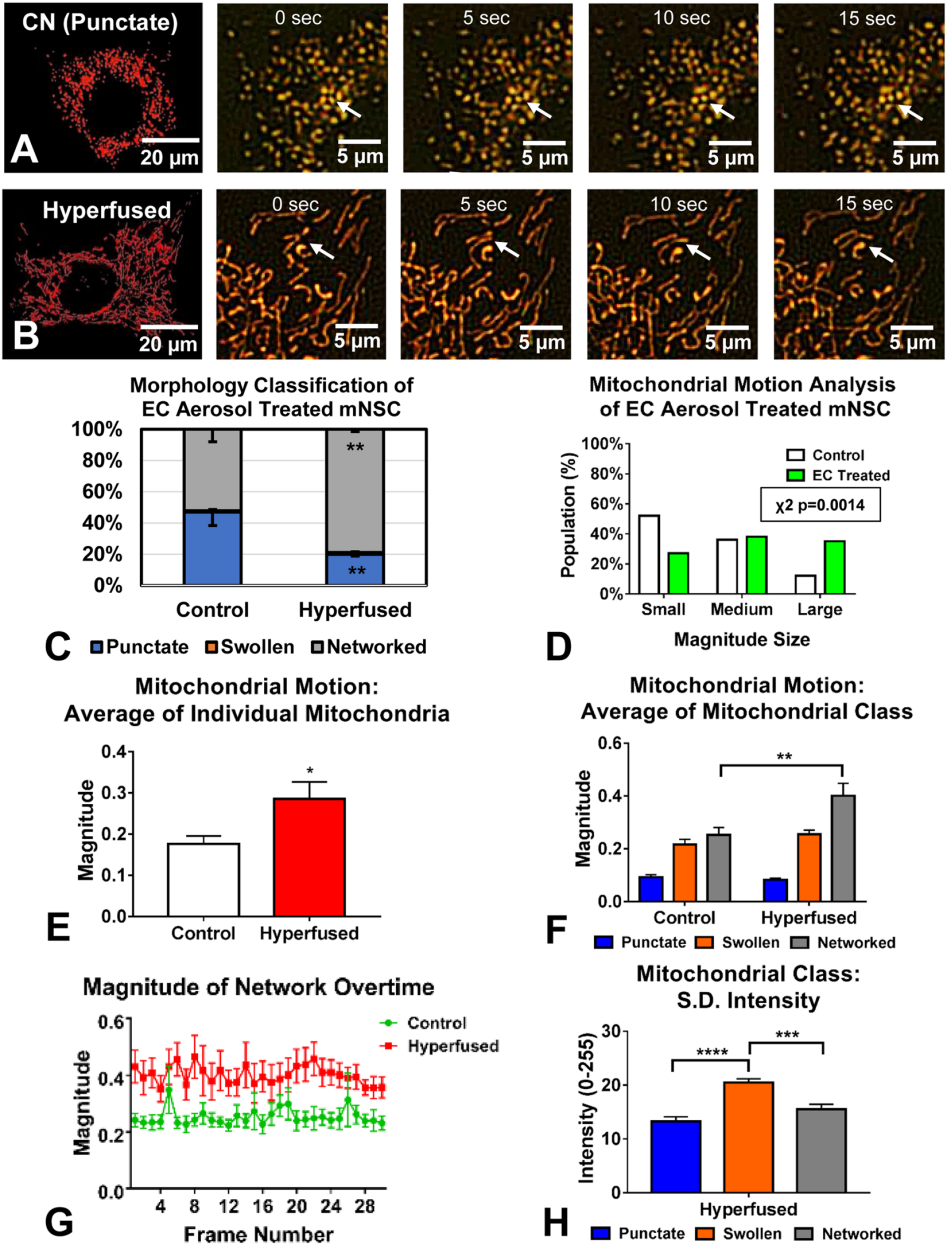
Analysis of stress-induced mitochondrial hyperfusion (SIMH) in NSCs. (**A**,**B**) Control NSC had primarily punctate mitochondria, whereas NSC treated with a tobacco product aerosol exhibited SIMH. Consecutive frames taken over 15 seconds show an enlarged section of live cells over time; arrows show examples of localized motion in the control and the translocation of hyperfused mitochondria. (**C**) Morphological analysis confirmed a significant shift towards the networked (hyperfused) morphology in the treated group. An unpaired two-tailed t-test was used when each morphological group was compared between the control and the hyperfused mitochondria (**D**) Motion analysis at the whole cell level showed an increase in motion in the hyperfused, treated mitochondria (p = 0.0014 using Chi-squared statistical analysis with a 95% confidence interval). (**E**) Motion analysis of individual networks of hyperfused mitochondria revealed a similar increase in motion (unpaired two-tailed t-test). (**F**) Motion analysis at the morphological class level showed that this increase in motion was due to the networked subgroup (2-way ANOVA with Sidak’s multiple comparisons test). (**G**) Magnitude of motion of the networked subgroup was greater than the control group over time. (**H**) Mean standard deviation of intensity analysis was performed for the three morphological classes in the hyperfused condition, showing that the swollen subgroup had the highest fluctuations in intensity (1-way ANOVA with Bonferroni’s multiple comparisons test).

Environmental toxicants can have a negative impact on mitochondria and alter their morphology^33^. To test the ability of MitoMo to detect changes in mitochondrial morphology and motion, NSC were treated with aerosol from an electronic cigarette that caused SIMH (Fig. 5A,B). The shift from a punctate to networked morphology was first confirmed using the morphology classifier in MitoMo (Fig. 5C). A statistically significant increase in networked mitochondria was detected in the hyperfused group using an unpaired two-tailed t-test. When examined microscopically, the hyperfused mitochondria did not have much motion. However, MitoMo motion analysis of the entire mitochondrial population showed a significant increase in motion in the electronic cigarette-treated, hyperfused mitochondria (p = 0.0014 for Chi-squared analysis with a 95% confidence interval) (Fig. 5D). This pixel-based analysis method is particularly powerful since it is otherwise not possible to quantify motion in the hyperfused mitochondria, which are no longer individual trackable objects. When motion was analyzed using unpaired two-tailed t-test at the level of individual mitochondria and then averaged, a significant increase in motion was observed in the hyperfused group (Fig. 5E).

To further isolate the increased motion to the hyperfused class, MitoMo was used to compare motion heterogeneity in morphological classes of mitochondria. The punctate population exhibited the least motion, followed by the swollen, and the networked class (Fig. 5F). When analyzed using a 2-way ANOVA with Sidak’s multiple comparison test, the magnitude of motion was greater in the networked mitochondria of the hyperfused group than in the untreated control (Fig. 5F). This increased movement could be due to the intermixing of mitochondrial membrane proteins, which is consistent with occurrence of SIMH to rescue damaged mitochondria^34^.

When the magnitude of motion of the networked subgroups was assessed over time, the hyperfused mitochondria had a consistently higher level of motion than mitochondria in control cells (Fig. 5G). To determine how intensity differed across the three morphological classes in the hyperfused condition, the mean standard deviation (S.D.) of the intensity was computed, revealing a statistically significant elevation in fluctuations of the intensity of the swollen mitochondria (Fig. 5H). This shows greater variation in sub-organelle intensity within the swollen mitochondria, which may be due to increased structural disorganization allowing greater movement of proteins.

### Characterization of the mechanism of selenium-induced mitochondrial swelling

**Figure 6:**
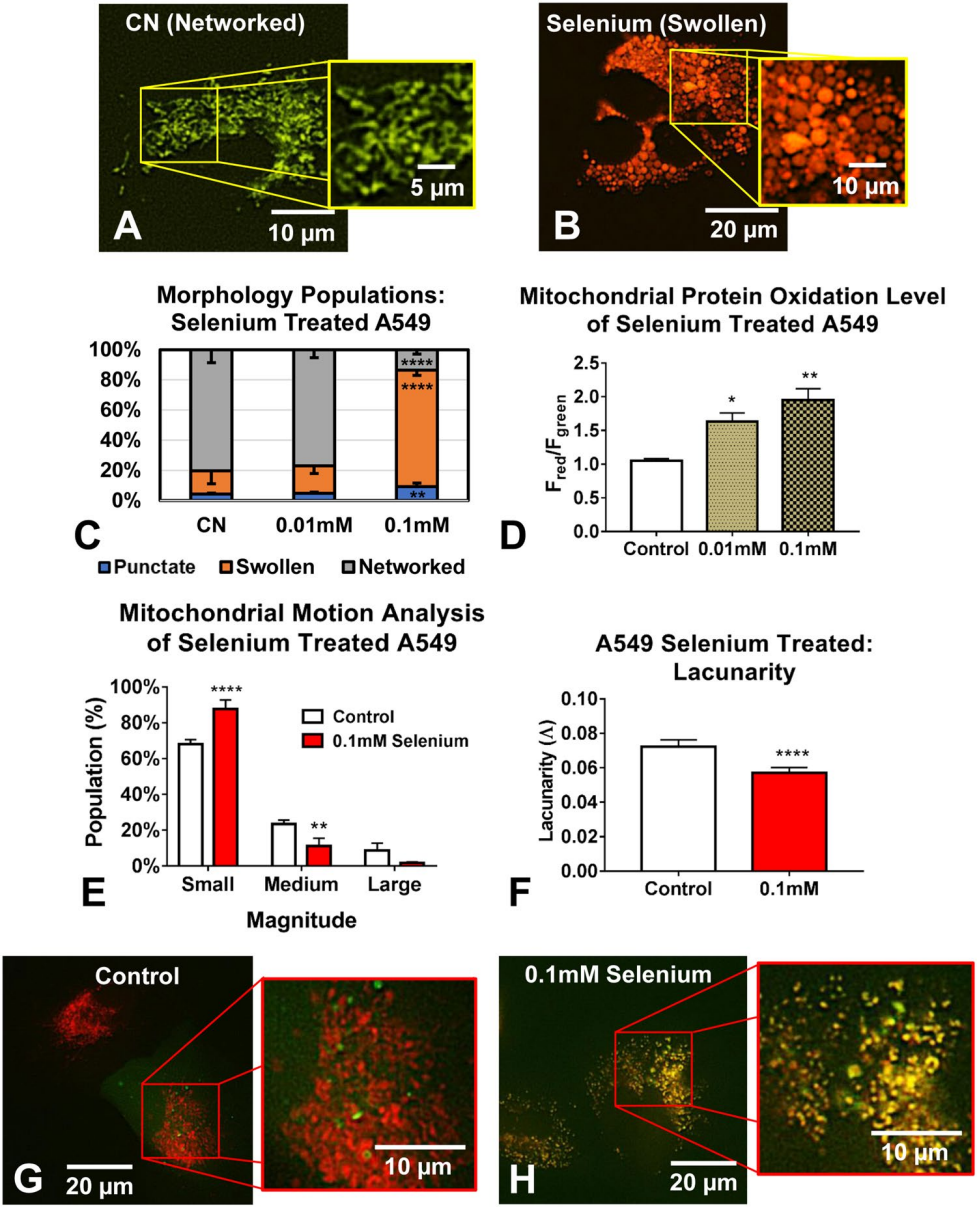
Analysis of swollen mitochondria in selenium treated A549 cells. (**A**,**B**) Treatment of MitoTimer-transfected A549 cells with 0.1 mM SeCl_4_ changed mitochondrial shape from networked to swollen and increased mitochondrial protein oxidation (increase in orange/red fluorescence). (**C**) Selenium treatment cells produced a dose dependent increase in the swollen mitochondria (1-way ANOVA with Dunnett’s multiple comparisons test). (**D**) The MitoTimer red/green fluorescence ratio showed a dose-response increase in mitochondrial protein oxidation in the selenium treated A549 cells (1-way ANOVA with Dunnett’s multiple comparisons test). (**E**) Motion decreased in the swollen mitochondria in the selenium treated group (2-way ANOVA with Bonferroni’s multiple comparisons test). (**F**) Lacunarity decreased in the treated, swollen mitochondria (unpaired two-tailed t-test). (**G**,**H**) Double labeling with GFP-LC3 showed that the mitochondria in selenium treated cells, but not in control cells, were co-localized with autophagosomes, indicating their targeted degradation.

In another toxicological application of MitoMo, we analyzed the effects of selenium, a contaminant in some tobacco products^35,36^, on mitochondrial health. Lung A549 cells were transfected with the MitoTimer reporter (Fig. 6A) and treated for 24 hours with 0.01 mM or 0.1 mM selenium tetrachloride (SeCl_4_) (Fig. 6B). MitoMo analysis of mitochondrial morphology showed a significant increase in the swollen phenotype (Fig. 6C) at the 0.1 mM concentration, and a significant dose-dependent increase in the oxidation of mitochondrial proteins (Fig. 6D) (both statistical analyses were done using a 1-way ANOVA with Dunnett’s post hoc test). Motion analysis demonstrated a significant decrease in mitochondrial motility in selenium treated cells when compared to controls (2-way ANOVA with Bonferroni’s multiple comparisons test) (Fig. 6E). Texture analysis using an unpaired two-tailed t-test showed that lacunarity decreased, consistent with the denser organization caused by mitochondrial swelling (Fig. 6F). Double labeling with a GFP-LC3 (microtubule-associated protein light chain 3a) autophagy reporter and MitoTracker dye demonstrated that the swollen mitochondria co-labeled with autophagosomes, indicating their targeted degradation via mitophagy (Fig. 6G,H).

### Mitochondrial morphogenesis during swelling in selenium treated A549 cells

**Figure 7:**
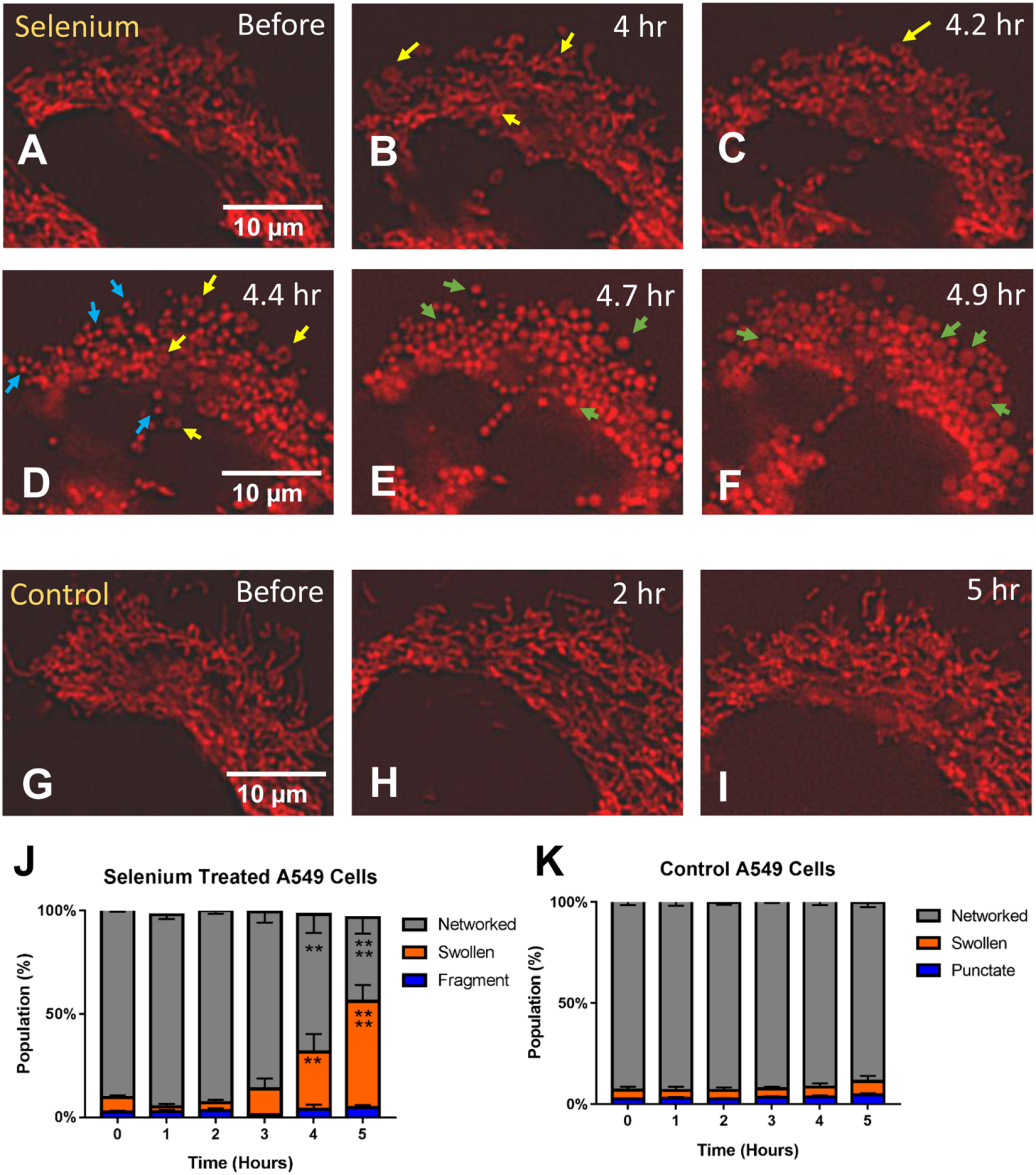
Mitochondrial morphogenesis in A549 cells during selenium treatment. (**A**–**F**) Time-lapse videos of 0.1 mM selenium-treated MitoTimer-transfected A549 cells were collected over several hours. The networked mitochondria first formed small tubes and donuts (yellow arrows in **B**–**D**), followed by fragmentation into the punctate morphology (blue arrows in **D**), before eventually expanding to form the swollen phenotype (green arrows in **E**,**F**) after approximately 5 hours of time. (**G**) The selenium-treated cells had an increase in the swollen mitochondria after 4 hours. (**H**) Control videos were also collected over several hours, and mitochondrial morphology did not change over time. Statistical analysis for (**G** and **H**) were done using a 2-way ANOVA with Dunnett’s multiple comparisons test.

MitoMo was used to analyze mitochondrial morphogenesis during treatment of A549 cells with 0.1 mM selenium in time-lapse videos collected every hour. By approximately 4 hours of treatment, mitochondrial morphology had changed from a primarily networked phenotype (Fig. 7A) to small rings (yellow arrows in Fig. 7B–D). Mitochondrial morphology progressed to the punctate phenotype (blue arrows in Fig. 7D), and approximately 1 hour later the punctate mitochondria expanded to form the swollen morphology (green arrows in Fig. 7E,F). Progression to the swollen morphology is shown quantitatively in the graphs for selenium-treated cells (Fig. 7J). Control cells (Fig. G–I) were also imaged every hour, and their morphologies remained primarily networked over time (Fig. 7K). Statistical analysis of both the treated and control groups was done using a 2-way ANOVA with Dunnett’s post hoc test.

### Health classification in cells using supervised learning

**Figure 8:**
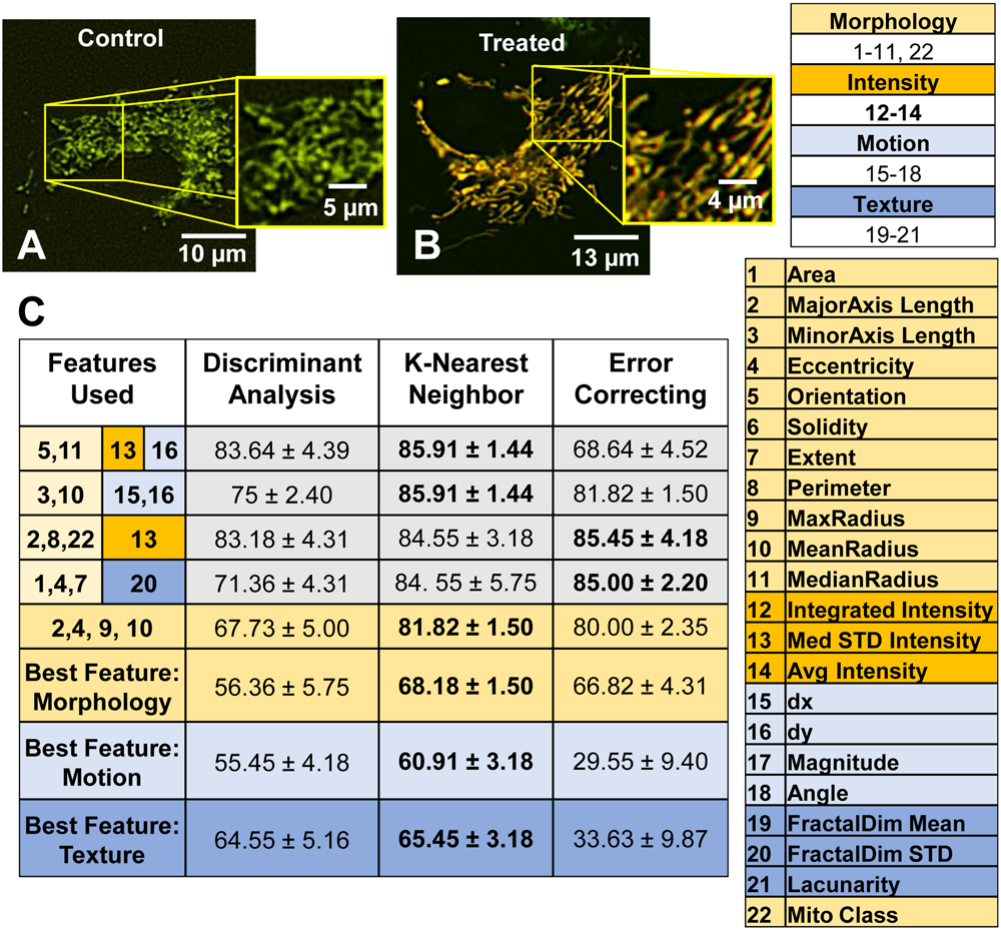
Health classification of A549 cells in “Undetectable stress” conditions. (**A**,**B**) Treatment of MitoTimer-transfected A549 cells with 0.01 mM SeCl_4_ did not visibly change their networked morphology (**C**), however, health classification could distinguish treated from control cells by combining different feature types. (**C**) Using a single morphology, motion, or texture feature at best resulted in a 68% accuracy in distinguishing the control and treated groups. Combining feature types resulted in up to 86% accuracy for KNN, 85.5% for error correcting method, 83.5% for discriminant analysis. Morphology features are numbered 1–11, and 22 and highlighted in light yellow. Intensity features are numbered 12–14 and highlighted in dark yellow. Motion features are numbered 15–18 and highlighted in light blue. Texture features are numbered 19–21 and highlighted in dark blue.

A549s cells treated with 0.01 mM of selenium were segmented after 24 hours of incubation using their MitoTimer intensity (Fig. 8B). When looking at morphology alone, it was extremely difficult to distinguish the controls from the low-dose treated cells, since both exhibited highly networked mitochondria. Therefore, all 21 morphology, intensity, texture, and motion features were tested either singly or in combination to distinguish the control and treated groups using three classifier algorithms: discriminant analysis^37^, K-Nearest Neighbor (KNN)^29^, and error correcting^38^ (Fig. 8C). KNN outperformed the other two classifiers in most cases, although the error correcting method had the potential to do well when there were multiple features. One feature alone resulted in maximum accuracy of 68% for morphology, 61% for motion, and 65% for texture. By combining four features, we were able to increase accuracy to 86%. This demonstrates that MitoMo allows accurate classification of cellular stress that was not distinguishable by visual inspection of mitochondrial morphology.

## Discussion

Machine learning and automated analysis of still and video data are important components of modern light microscopy^39^. Software is needed that has the capability of extracting biologically relevant data from images in a reliable, unbiased, and rapid manner. MitoMo has a user-friendly interface, is open source (see Methods), and could be used to analyze any type of mitochondrial morphology given user-input trained libraries. Our segmentation is versatile (allows import of pre-segmented images) and has tunable image processing parameters allowing for high accuracy. While other mitochondrial programs often analyze a limited number of features^15,16,19–23,26^, MitoMo enables a deep level of analysis that integrates morphology, texture, motion, morphogenesis, and cell health classification into a single program. This is important, since features that appear uninformative by themselves can be highly relevant if combined with other features. MitoMo is the only machine-learning software that provides both mitochondrial morphological classification and cell health classification based on mitochondrial features. MitoMo incorporates both still and video image analysis making morphogenesis and dynamic analyses possible with a single software program. While our data were analyzed using deconvoluted fluorescent images, confocal, super resolution and holotomographic images could also be used with MitoMo and may be easier to segment given their higher resolution.

Mitochondria have been used to study a variety of biological problems such as stress response^22,23^, drug response^16,18,22,40,41^, classifying cell types^19^ and disease states^15,21^. MitoMo has all of these analytical capabilities, but with a greater number of feature types allowing deeper analysis. For instance, when morphology alone was not adequate to distinguish different cell types, clear segregation of cells types was possible with texture analysis (Fig. 3I). This was shown in the case of hPFs and A549s, where both exhibited networked mitochondria but could be distinguished using texture features. Previously, stress responses were studied using morphology (e.g. hyperfusion or mitophagy-induced morphological changes). For example, we and others have shown that stressors such UV, antibiotics, and environmental toxicants can cause mitochondrial hyperfusion^6,8^. Here, we show that stress-induced mitochondrial hyperfusion is also accompanied by an increase in motion.

Changes in mitochondria morphology and dynamics can correlate with mitochondrial function, as well as with various types of stimuli. For instance, mitochondria are transported to sites of energy demand^42^ and changes in mitochondrial morphology correlate with permeability and respiratory functions^24^. Here, we showed that stress induced mitochondrial hyperfusion occurred in NSCs treated with electronic cigarette aerosol. We know from previous observations that this survival response correlates with increased production of ATP^6,8^, a hallmark function of mitochondria, as well as an increase in ROS, which could be damaging to the cell. Since NSC are critical for post-natal brain development^43^, stress to their mitochondria would be a concern for normal functioning of these cells and corresponding tissue. Moreover, we show that response to EC aerosol-induced stress results in an increase in mitochondrial protein oxidation using intensity feature analysis of MitoTimer reporter. MitoMo goes beyond traditional intensity analysis to include features to measure how intensity values fluctuate (higher in swollen mitochondria, shown in Fig. 5H), which expand intensity analysis to the study of dynamics. Any mitochondrial-targeted dye or reporter could be used with MitoMo to quantify functional readouts.

MitoMo’s novel intensity flow method for motion analysis bypasses the limitations of traditional object tracking and optical flow motion analysis. Because MitoMo uses a pixel-based method, it is particularly powerful at quantifying mitochondrial motion when mitochondria are not trackable (e.g., when they exist in networks). Also, when movement of entire mitochondria is arrested (as in the case of selenium-induced toxicity), motion within the mitochondria can still be assessed using MitoMo. Also, MitoMo circumvents the brightness over time constraint of optical flow, resulting in significantly higher angle and magnitude accuracies in 4 out of 6 synthetic motion conditions. Also, our software can evaluate direction of both intact mitochondria with respect to any cellular structure and directional motion within the mitochondria. Directional motion of mitochondria can be useful when mitochondria are reorganized, such as during perinuclear clustering, which could be an early biomarker of cell stress^44,45^. In addition, directional analysis can be used to detect motion heterogeneity in mitochondrial distribution and translocation inside a cell. MitoMo was used to show direction of movement to/away from the nucleus and alteration of that movement following drug treatment. Although not shown in this paper, the ability of MitoMo to analyze intra-organelle motion can be applied to show flow of mtDNA or specific mitochondrial proteins.

MitoMo was able to evaluate morphogenesis of mitochondrial swelling and eventual mitophagy, which occurred during selenium-induced stress^46–48^. Selenium has been implicated in mitochondrial toxicity^49,50^ and is found in some tobacco products, including electronic cigarette fluids and aerosols^35,36^. A549 cells treated with selenium transitioned from the networked to punctate morphology consistent with fission, a process that is needed to segregate dysfunctional mitochondria for culling by mitophagy^51^. The punctate mitochondria then became swollen, likely due to increases in mtROS^52^ (shown in Fig. 6B,D). This is consistent with inhibition of the respiratory chain and the loss of mitochondrial membrane potential (MMP) following metal or selenium treatment^53^. Our data provide clear evidence that treatment of a cancer cell line with 0.1 mM selenium adversely affects mitochondria and may be a mechanism contributing to treatment of cancer with selenium^54^. It will be important in future work to determine if selenium has similar adverse effects on normal cells, as such effects would impair health.

Phenotyping with MitoMo also provides the information needed to classify healthy and unhealthy cells. As shown previously, mitochondrial phenotyping based on texture can distinguish different types of cancer cells and their response to drugs^15,16,21^. As shown in Fig. 8, health classification with MitoMo can identify subtle changes in mitochondria and was useful in discovering stress that was not detectable by examining mitochondrial morphology, texture, or motion alone. Subtle sub-organelle changes have traditionally been observed using ultra-high resolution techniques, such as transmission electron microscopy (TEM)^55,56^; however, mitochondrial analysis at this scale does not allow for whole-cell phenotyping, is extremely time-consuming, and is limited to fixed cells. Early detection of mitochondrial stress has many applications including high-throughput drug testing, toxicology, and any biological research involving mitochondria.

Mitochondrial health plays an important role in many diseases, including neurodegenerative diseases such as Parkinson’s, Huntington’s, and Alzheimer’s^11,57^, metabolic disorders^7^, cancer^54,58,59^, and mitochondria myopathies^11,57^. Although not shown in this paper, MitoMo could be used to identify early biomarkers of specific disease by deep analysis of mitochondria from easy to obtain human biopsies, such as blood, urine, saliva, and skin. Moreover, there are numerous applications of MitoMo to disease-in-a-dish models in which changes in mitochondrial morphology, motion, and texture could be studied against control cells during disease progression. MitoMo could also be used to study the morphology and dynamics of other organelles, such as peroxisomes and autophagosomes/lysosomes, which have important links to disease. MitoMo can be used with any cell type and expanded to study the dynamics of any organelle.

In summary, MitoMo has immediate and broad utility in cell biology, drug discovery, toxicology, and medicine in that it permits deep quantitative evaluation of mitochondrial morphology, motion, texture, morphogenesis, and cell health classification in an accessible integrated software package.

## Materials and Methods

### Cell culturing and reagents

NSC (line C17.2, provided by Dr. Evan Snyder’s lab) were grown as previously described^6^. A549 lung epithelial cells (human type II pulmonary alveolar adenocarcinoma cells) were obtained from ATCC. Cells were cultured in F-12K medium (Kaighn’s Modification of Ham’s F-12, ATCC #30-2004) and 10% fetal bovine serum (ATCC #30-2020). Cells were incubated at 37 °C in 5% CO_2_ until 80% confluency, at which point they were detached using 0.25% trypsin. Cells were passaged every 2–3 days and medium was replenished every other day. Human pulmonary fibroblasts (hPF) (obtained from ScienCell) were cultured in complete fibroblast medium (ScienCell) containing 2% fetal bovine serum, 1% fibroblast growth serum, and 1% penicillin/streptomycin. hPF were grown on poly-L-lysine (2 μl/1 ml) coated T-25 flasks, which were prepared a day in advance of use in experiments. hPF were cultured in 5% CO_2_ at 37 °C and 95% relative humidity until 70–80% confluent. For sub-culturing and experimental set up, cells were washed with DPBS and detached with 0.05% trypsin diluted in DPBS for 1 minute at 37 °C.

### Live cell imaging

For live imaging, cells were plated on µ-Slide Ibidi 8-well chambers (Ibidi) at approximately 6,000 cells/well. Time-lapse fluorescent images were collected using a TI inverted Nikon Eclipse microscope equipped with a LiveCell temperature and CO_2_-regulating, heated stage (Pathology Devices Inc). The images were collected using a Nikon 60 × 0.85 NA objective with a 0.11 um/pixel resolution, and a Nikon 40 × 0.75 NA and 60 × 1.4 NA objectives with 0.16 and 0.11 µm/pixel resolutions respectively. A high-resolution Andor Zyla VSC-04941 camera (Andor, Belfast, UK) was used. Excitation illumination was from the Nikon INTENSILIGHT C-HGFIE lamp. Videos were collected at millisecond resolution and deconvoluted using the “live de-blur” feature of the NIS Elements software (Nikon).

### Transiently transfected and reporter cells and labeling of mitochondria

NSC were cultured, detached enzymatically, and centrifuged for 3 minutes at 3,000 rpm. The pellet containing 6 × 10^6^ cells was re-suspended in Nucleofection medium (Lonza Kit #VAPG-1004) and nucleofected in the Amaxa Nucleofector IIb device (Lonza) using program A-033. The cells were quickly transferred to a new dish containing pre-equilibrated fresh medium, and selected with hygromycin at an optimal kill dose of 150 µg/ml. The medium and hygromycin treatment was replenished every other day for 30 days. The Mito-Timer-transfected NSC were further enriched with FAC Sorting (FACS Aria) using GFP (488 nm Argon laser, 530/30 filter) and DsRed (488 nm Argon laser, 610/20 filter) signals. A549 and hPF cells were transiently transfected using DNA-In reagents (MTI-GlobalStem #73770 and 73750). The Addgene plasmids (pMitoTimer #52659 and pEGFP-LC3 #21073) were used to transfect the cells. A549 cells were co-labeled with MitoTracker-Red dye (ThermoFisher Scientific #M7512) to visualize mitophagy.

### Quantification of mitochondrial texture and motion using MitoMo

By computing the change in intensity of fluorescently-labeled mitochondria between adjacent frames, the flow of mitochondrial proteins can be estimated at the individual pixel level. To do this, a difference image *D*_*t*,*t+1*_ was computed as,

$${D}_{t,t+1}={I}_{t+1}-{I}_{t},$$

where *I*_*t*_ is the image at time *t*. Negative values in *D*_*t*,*t*+1_ represent a decrease in intensity at that pixel location, while positive values represent an increase. *D*_*t*,*t*+1_ shows a change in the density of fluorescent tags over time. To visualize these changes, *D*_*t*,*t*+1_ is rescaled with the equation,

$${D}_{t,t+1}^{^{\prime} }=\frac{{D}_{t,t+1}+255}{510},$$

so that the pixel values are between 0 and 1. To filter out background motion and motion in other structures, a region of interest is estimated for each difference image. MitoMo used an adaptive Otsu’s segmentation^60^ on each frame to segment the structures of interest. The region of interest for each difference frame is defined as,

$$RO{I}_{t,t+1}={S}_{t}\cup {S}_{t+1},$$

where *S*_*t*_ is Otsu’s segmentation output at time t. Using ${D}_{t,t+1}^{^{\prime} }$ and *ROI*_*t*,*t*+1_, motion vectors in the image sequence are computed with the following gradient equation:

$$\nabla {D}_{t,t+1}^{^{\prime} }=\frac{\delta {D}_{t,t+1}^{^{\prime} }}{\delta x}\hat{x}+\frac{\delta {D}_{t,t+1}^{^{\prime} }}{\delta y}\hat{y}=u\hat{x}+v\hat{y}.$$

$\hat{x}$ and $\hat{y}$ are the unit vectors corresponding to the x and y axes. The equation above does not rely on the brightness assumption of optical flow and instead examines the gradient of intensity. The magnitude and angle of the motion vectors are calculated as follows,

$$\begin{array}{rcl}M & = & \sqrt{{v}^{2}+{u}^{2}}\\ \theta  & = & {\tan }^{-1}\frac{v}{u}\end{array}$$

While vectors can be analyzed individually, they may be combined over a region of interest. For every mitochondrion, vector addition is performed on the vectors that lay within the segmented mitochondrion. For the whole-cell level, vector addition is performed on all the vectors within the cell. This produces a single vector representing the motion of that mitochondria or cell. In addition to the generated motion vectors, other features are extracted for each level of analysis. While basic morphological and intensity features are examined, texture features are of interest as they can analyze density of visual patterns. A differential box counting method was used to compute the fractal dimension^61^ of a region around a pixel. Fractals are self-repeating patterns whose complexity can be represented by their dimension and density by their lacunarity^62^.

### Segmentation and classification of mitochondrial morphology using MitoMo

MitoMo was used to segment the red fluorescent channel from MitoTimer-transfected cells. Pre-segmented images were also imported from CellProfiler software. Histogram matching^63^ was performed on each video using a reference video to control intensity levels. Thresholding was performed using a global Otsu’s method^60^, and morphological, intensity, texture, and motion features were extracted for each segmented mitochondrion. Because segmented regions may be clustered together after thresholding, a watershed algorithm^64^ was used to de-clump the segmented regions. This is done by finding the regional maximal intensities of each segmented region and computing a boundary that best separates the maxima. The extracted features were fed to supervised learning algorithms KNN and Naïve Bayes written on the MATLAB platform. The software was trained with image libraries of each morphological type (punctate, networked, swollen). An exhaustive search was carried out to identify key features and combinations that allowed accurate morphology-based classification. The experimental datasets were analyzed by the classifiers to determine the total area of each morphological subpopulation, which was normalized by the total mitochondrial area in each cell and averaged across all cells within each group.

### Health classification of “stressed” cells

MitoMo was used to segment the red fluorescence channel from MitoTimer-transfected cells. Histogram matching was done for every frame of the video with reference to the “before” (Time = 0) video of the dataset, and segmentation was performed. Since classification of stressed cell conditions is done at the video scale, video features are found by computing the mean (average) of the feature value over the entire length of video. These features were previously extracted for mitochondrial analysis. Morphology, intensity, texture, and motion features were tested either singly or in combination to distinguish the two treatment conditions using three classifier algorithms: discriminant analysis, K-Nearest Neighbor (KNN), and error correcting output codes classifier.

### Statistical analysis

For each set of data, three independent experiments were performed. Data were graphed and analyzed statistically using Prism software (GraphPad) as described in the figure legends. Data were considered to be significantly different for p < 0.05.

### Source Code and Executable Files

MitoMo was written and developed with MATLAB 2018a. The source code and an executable GUI are available online at http://vislab.ucr.edu/SOFTWARE/software.php. MitoMo.m is the main file of the code and requires the following MATLAB toolboxes: System Identification, Image Processing, Statistics and Machine Learning, and Bioinformatics. The executable requires the installation of the 64-bit version of MATLAB Runtime R2018a (9.4) available at http://www.mathworks.com/products/compiler/mcr/.

## Electronic supplementary material

Supplementary Information

## Data Availability

All relevant data are available within the manuscript. The source code for MitoMo and an executable GUI are available at http://vislab.ucr.edu/SOFTWARE/software.php.
