# Supplementary material for: Deep Analysis of Mitochondria and Cell Health Using Machine Learning
Source: Sci Rep. 2018 Nov 5;8:16354. doi: 10.1038/s41598-018-34455-y (PMC6218515; doi:10.1038/s41598-018-34455-y)
Supplement: Supplementary file 1 — Supplementary Information [file 41598_2018_34455_MOESM1_ESM.pdf]

Deep Analysis of Mitochondria and Cell Health Using Machine Learning

Atena Zahedi[1,3], Vincent On[2], Rattapol Phandthong[3], Angela Chaili[3], Guadalupe Remark[3], Bir Bhanu[2,1], Prue Talbot[1,3]*

[1]Graduate Program in Bioengineering, University of California, Riverside, CA. USA

[2] Department of Electrical & Computer Engineering, University of California, Riverside, CA. USA

[3] Department of Molecular, Cell and Systems Biology and Neuroscience, University of California, Riverside, CA. USA

*Corresponding author

Prue Talbot

Running Title: Deep analysis of Mitochondria

Supplementary Information

Supplemental Data #1: A complete list of extracted features.

1. Area – Number of pixels in a segmented region.
2. Major Axis Length – Number of pixels on the major axis of the fitted ellipse.
3. Minor Axis Length – Number of pixels on the minor axis of the fitted ellipse.
4. Eccentricity – Ratio of the distance between the foci of the ellipse to its major axis length.
5. Orientation – Angle between major axis of ellipse and the x-axis.
6. Solidity – Ratio of the area of the segmented region over the area of the convex hull of the segmentation.
7. Extent – Ratio of the area of the segmented region over the area of the bounding box of the segmented region.
8. Perimeter – Number of pixels in the contour of the segmented region.
9. Max Radius – The maximum distance of any pixel in the segmented region to the closest background pixel.
10. Mean Radius – Mean value the distances of all pixels in the segmented region to their closest background pixel.
11. Median Radius – Median value the distances of all pixels in the segmented region to their closest background pixel.
12. Average Intensity – The average of pixel intensities within the segmented region.
13. Integrated Intensity – The sum of the pixel intensities within the segmented region.
14. Median Standard Deviation of Intensity – The median of standard deviation of intensity values in a segmented region. These values are obtained by computing the standard deviation of the intensity values of a pixel across all frames of a video.
15. dx – The x component of a motion vector, which can be computed at either a pixel or mitochondrion level.

16. dy – The y component of a motion vector, which can be computed at either a pixel or mitochondrion level.
17. Magnitude – The magnitude of a vector, which can be computed at either a pixel or mitochondrion level.
18. Angle – The angle of a vector, which can be computed at either a pixel or mitochondrion level.
19. Fractal Dimension Mean – The mean value of the fractal dimensions of each pixel in a segmented region. Higher values represent more complex patterns.
20. Fractal Dimension Standard Deviation – The standard deviation of the fractal dimensions of each pixel in a segmented region.
21. Fractal Dimension Lacunarity – The squared value of the standard deviation of fractal dimension divided by the mean fractal dimension. Lower value represents a dense pattern while higher values represent more open patterns.
22. Mitochondria Morphological Class: The punctate, swollen, and networked morphologies.

Supplemental Data #2: Segmentation, morphological, and motion validation of MitoMo.

Ground-truth analysis was performed to verify the accuracy of mitochondrial segmentation with both MitoMo and CellProfiler by comparing segmented images with mitochondria manually traced using ImageJ. The percent overlay of both the CellProfiler and MitoMo segmentations was not statistically different than the ground-truth (one-way ANOVA with Dunnett's post hoc test). Next, ground-truth analysis was done to validate morphology classification by manually labeling mitochondria as punctate, networked, or swollen. This ground-truth was used as the training library. Next, the software's classifier, which was up to 88% accurate using the Naïve Bayes classifier, was used to automatically categorize the mitochondria.

To validate motion analysis, various synthetic shapes with motion were created using a script written in MATLAB (Figs. 2g-l). Two shapes were used, a disk with a radius of 5 pixels and

an 8 by 15-pixel rectangle. Each shape was tested on three conditions to simulate intensity changes in fluorescence images: a solid shape of uniform intensity, a shape with varying intensity that has higher brightness towards the center, and a fading shape with varying intensity which losses half of its brightness from the first to second frame. Each shape appears in the center of a 30 by 30-pixel image with a black background and is moved to another position by a random distance and direction in the next frame. A random motion with a maximum of 3 pixels was applied to the object in both the x and y directions. Motion magnitude and angle accuracy were tested 10 times, each time using 1000 random synthetic configurations for the 6 conditions (Fig. 2M). The mean and standard deviation for angle and magnitude values were computed from this randomly generated motion. Since ground-truth and the generated magnitudes of our motion vector and optical flow vector were at different scales, these values were max-min normalized so that all values were between 0 and 1. The estimated angle of motion was assessed to be correctly generated if it was within 30 degrees of the ground-truth angle. Magnitude was tested separately in the x and y dimensions to avoid rescaling issues. Similarly, if both the x and y magnitudes of the generated vector were within 33% of the ground-truth, it would be considered correctly estimated. MitoMo was compared against the Lucas-Kanade method for computing optical flow, and percentage of accuracies are summarized in the table in Figure 2M. Statistical analysis of each condition was conducted using a two-tailed t-test and Chi-squared test in cases where there was no variance. The more accurate software is highlighted in yellow in the table.
